# Strategic testing plan for ambulatory surgery centers after the COVID-19 pandemic

**DOI:** 10.1017/dmp.2020.426

**Published:** 2020-11-04

**Authors:** Ramana Naidu, Samir Sheth, Rahul Chaturvedi, Krishnan Chakravarthy

**Affiliations:** 1 California Orthopedics & Spine, Larkspur, California, USA; 2 Sutter Roseville Pain Management, Roseville, California, USA; 3 Division of Pain Medicine, Department of Anesthesiology, University of California San Diego, La Jolla, California, USA; 4 VA San Diego Health Care, San Diego, California, USA

**Keywords:** emergency medical services, health impact assessment, infectious disease medicine, public health practice, quality of health care

## Abstract

As the curve continues to flatten during the severe acute respiratory coronavirus 2 (SARS-CoV-2) pandemic, and more physicians resume outpatient clinical work, the question arises of how to ensure the safety of the patients and staff while performing cases. Many institutions and health-care offices have turned to screening questionnaires to determine the likelihood of coronavirus disease 2019 (COVID-19) positivity. However, screening questionnaires are woefully inadequate as studies have shown that roughly 6.4% to 50% of patients may spread this virus without any symptoms. In this study, we have outlined a proposal to restart elective procedures after the curve has flattened in a certain locale, particularly for ambulatory surgery centers (ASCs). Until additional data are collected for specific sensitivity and specificity values for PCR testing, we recommend performing 2 consecutive polymerase chain reaction (PCR) tests to minimize false negative rates. The algorithm described in this study can help ASCs begin their practices and provide local public health officials with valuable data that can help establish true sensitivity and specificity rates for these tests.

The coronavirus disease 2019 (COVID-19) global pandemic, caused by severe acute respiratory syndrome coronavirus 2 (SARS-CoV-2), has brought on unprecedented and unpredictable changes to our everyday society. Although it may be possible to close nonessential services to stop the spread of this highly contagious virus, essential services must continue. During the initial stages of the COVID-19 crisis, the surgeon general called for postponing all elective surgeries, and since then, many states have made strong recommendations regarding elective cases, especially with regard to ambulatory surgery centers.^1^ Moreover, the American College of Surgeons has also presented physicians and surgeons with helpful guidance regarding the classification of procedures and surgeries,^2^ but truly defining elective surgeries with regard to pain management causes considerable confusion.^3^ What is deemed elective initially may become more urgent with every week or month that passes under quarantine.

Recently, the American Society of Regional Anesthesia (ASRA) developed valuable guidelines to help manage pain patients during this time, but these guidelines do not provide risk mitigation strategies to bring patients to the surgery center safely.^4^ As we continue to flatten the curve and begin the process of resuming clinical work, the question arises of how to ensure the safety of the patients and the staff while performing these cases. Many institutions and health-care offices have turned to screening questionnaires to determine the likelihood of COVID-19 positivity. However, screening questionnaires are woefully inadequate as studies have shown that roughly 6.4% to 50% of patients without any symptoms may spread this virus.^5,6^ Government policies have correctly adopted physical and social distancing programs. However, while isolation policies may be effective in decreasing the viral spread of COVID-19, best case scenarios estimate the need for at least 3 mo of continued isolation.^7^ Given the ever-growing number of patients who have been left untreated due to their procedures being deemed “non-essential,” ambulatory surgery centers (ASCs) will experience a rapid surge of patients as soon as the curve flattens. However, even after the curve has flattened, the risk of transmission still exists, and the specter of a second peak as seen in many American cities during the 1918 H1N1 pandemic could occur.^8^ One solution that would allow for the safety of patients and for the safety of those taking care of them would be to test every patient before surgery at the ASCs. The idea would be to make every ASC a “coronavirus free zone,” or CFZ.

Unfortunately, testing has not been uniformly available in the United States, and there is also concern for a high false negative rate in the tests that are being run.^9^ While sputum samples may decrease the rate of false negatives and should be considered in hospital and outpatient urgent care settings, the length of time to run the test may pose an issue for patients needing to be “cleared” that day for surgery.^10^ Under the Emergency Use Authorization (EUA) by the Food and Drug Administration (FDA), many rapid tests have been developed. We have provided a comprehensive summary of the available tests as seen in Table 1.

**Table 1.** FDA approved emergency use authorization qSARS-CoV-2 PCR and serology testing kits

Test	Company	Cost of Test	Cost of Machine	Type	Method	PPA	N	95% CI	NPA	N	95% CI	Speed of Results
ID NOW COVID-19	Abbott	$40/cartridge	$50,000	PCR	Nasopharyngeal swab	100	30	(88.7, 100)	100	30	(88.7, 100)	5 min
qSARS-CoV-2 IgG/IgM Rapid Test	Cellex			ELISA	Serology	93.8	128	(88.2, 96.8)	96	250	(92.8, 97.8)	20 min
Accula SARS-Cov-2 Test	Mesa Biotech			PCR	Respiratory (multiple)	100	30	(88.7, 100)	100	30	(88.7, 100)	30 min
BioFire COVID-19 Test	BioFire Defense, LLC			PCR	Nasopharyngeal swab	100	30	(88.7, 100)	100	66	(94.5, 100)	45 min
Xpert SARS-CoV-2	Cepheid	$20/test	$175,000	PCR	Nasopharyngeal swab	100	30	(88.7, 100)	100	35	(90.1, 100)	45 min
Simplexa COVID-19 Direct	DiaSorin Molecular			PCR	Nasopharyngeal swab	100	52	(93.2, 100)	100	56	(93.6, 100)	60 min
COVID-19 genesig Real-Time PCR Assay	Primerdesign			PCR	Oropharyngeal swab	96	50	(8.3, 99.5)	100	50	(92.9, 100)	60 min
QIAstat-Dx Respirator SARS-CoV-2 Panel	QIAGEN GmbH			PCR	Nasopharyngeal swab	100	30	(85.8, 100)	100	30	(85.8, 100)	60 min
Lyra SARS-CoV-2 Assay	Quidel			PCR	Nasopharyngeal swab	100	92	(96.0, 100)	100	92	(96.0, 100)	75 min
NeuMoDx SARS-CoV-2 Assay	NeuMoDx			PCR	Nasopharyngeal swab	100	46	(92.2,100)	100	33	(89.5, 100)	80 min
Logix Smart Coronavirus Disease 2019 (COVID-19) Kit	Co-Diagnostics, Inc	$7/test		PCR	Unspecified	100	90	(95.6, 100)	100	90	(95.6, 100)	90 min
ePlex SARS-CoV-2 Test	GenMark Diagnostics			PCR	Nasopharyngeal swab	94.4	18	(74.2, 99.0)	100	47	(92.4, 100)	90 min
AvellinoCoV2 test	Avellino Lab USA, Inc			PCR	Respiratory (multiple)	100	30	(88.7, 100)	100	30	(88.7, 100)	1.5-3 h
BioGX SARS-CoV-2 Reagents for BD	Becton, Dickinson & Company			PCR	Nasopharyngeal swab	100	29	(88.1, 100)	100	30	(88.7, 100)	3 h
Panther Fusion SARS-CoV-2	Hologic			PCR	Nasopharyngeal swab	100	69	(94.7, 100)	100	109	(96.6, 100)	3 h
Real-Time Fluorescent RT-PCR Kit for Detecting SARS-2019-nCoV	BGI Genomics Co. Ltd			PCR	BAL	81	58	(69.1, 89.1)	100	165	(97.7, 100)	3 h
Real-Time Fluorescent RT-PCR Kit for Detecting SARS-2019-nCoV	BGI Genomics Co. Ltd			PCR	Oropharyngeal swab	91.2	34	(77.0,97.0)	100	67	(94.6, 100)	3 h
Cobas SARS-CoV-2	Roche			PCR	Nasopharyngeal swab	100	50	(92.9,100)	100	100	(96.3, 100)	3.5 h
NxTAG CoV Extended Panel Assay	Luminex Molecular Diagnostics Inc.			PCR	Nasopharyngeal swab	100	30	(88.7, 100)	100	30	(88.7, 100)	4 h
TaqPath COVID-19 Combo Kit	Thermo Fisher Scientific			PCR	BAL	100	30	(88.7, 100)	100	30	(88.7, 100)	4 h
TaqPath COVID-19 Combo Kit	Thermo Fisher Scientific			PCR	Nasopharyngeal swab	100	30	(88.7, 100)	100	30	(88.7, 100)	4 h
Abbott RealTime SARS-CoV-2	Abbott			PCR	Nasopharyngeal swab	100	60	(94.0, 100)	100	31	(88.8, 100)	1 d
COVID-19 RT-PCR Test	Lab Corp of America	$51.31/test		PCR	Nasopharyngeal swab	100	40	(91.2, 100)	100	50	(92.9, 100)	1 d
COVID-19 RT-PCR Test	Lab Corp of America	$51.31/test		PCR	BAL	100	40	(91.2, 100)	100	50	(92.9, 100)	1 d
Quest SARS-CoV-2 rRT-PCR	Quest Diagnostics	$50-$100/test		PCR	Respiratory (multiple)	100	30	(88.7, 100)	100	30	(88.7, 100)	1 d
COV-19 IDx assay	Ipsum Diagnostics, LLC			PCR	Nasopharyngeal swab	100	36	(90.4, 100)	100	30	(88.7, 100)	1 d
Gnomegen COVID-19 RT-Digital PCR Kit	Gnomegen LLC			PCR	Oropharyngeal swab	100	30	(88.7, 100)	100	30	(88.7, 100)	Unspecified
ScienCell SARS-CoV-2 Coronavirus Real-time RT-PCR (RT-qPCR) Detection Kit	ScienCell			PCR	Nasopharyngeal swab	100	30	(88.7, 100)	100	30	(88.7, 100)	Unspecified
PerkinElmer New Coronavirus Nucleic Acid Detection Kit	PerkinElmer, Inc			PCR	Oropharyngeal swab	100	47	(92.5, 96.2)	100	94	(96.2, 100)	Unspecified
PerkinElmer New Coronavirus Nucleic Acid Detection Kit	PerkinElmer, Inc			PCR	Nasopharyngeal swab	100	47	(92.5, 96.2)	100	94	(96.2, 100)	Unspecified
New York SARS-CoV-2 Real-time Reverse Transcriptase (RT)-PCR Diagnostic Panel	Wadsworth Center, New York State Department of Public Health’s (CDC)			PCR	Unspecified	97.7	43	(87.7, 99.9)	100	29	(88.3, 100)	Unspecified
CDC 2019-nCoV Real-Time RT-PCR Diagnostic Panel (CDC)	Centers for Disease Control and Prevention’s (CDC)			PCR	Respiratory (multiple)	100	13	(77.2, 100)	100	104	(96.4, 100)	Unspecified
1: Positive Percent Agreement 2: Negative Percent Agreement												

Abberviations: CI, confidence interval; NPA, negative predictive agreement.

## Solutions

We have outlined a proposal to restart elective procedures after the curve has flattened in a certain locale. This proposal is specific to freestanding ASCs as these facilities will experience a rapid surge in patients after the COVID-19 pandemic; additionally, after discussion with multiple directors of ASCs, these facilities may have the resources to execute this plan. The principles discussed in this proposal could also be applicable to a hospital or in-office setting.

### Know Your Data

To initiate elective surgery, it is important to know that the number of new infections has been declining for at least 14 d, and that the community’s health-care facilities have capacity. This suggests that the “curve may be flattening” and gives a degree of confidence that elective cases may begin. The primary caveat to this is that the data are only as good as the collection. Regions that do vigorous testing with high quality tests can provide a more accurate estimate than places that only test symptomatic patients. Review of these data are recommended to occur daily as new clusters may occur, thus prompting one to cease elective surgery.

### Communicate With the Local Department of Public Health

It is important to inform the local department of public health, municipality, or county, about the plans to start elective surgery. It provides a 2-way communication about cases and a deeper understanding of the data within local communities. This is a measure of goodwill that can strengthen a local community’s health-care system.

### Personal Protect Equipment Inventory

Ambulatory Surgery Centers across the United States have converted their mission from providing high-quality outpatient care to contributing health-care resources to the frontlines of those battling COVID-19. Personal protective equipment, or PPE, in the COVID-19 pandemic has been scarce in certain regions of the United States. Redistribution of such goods including other medical equipment such as ventilators, medications, or cleaning supplies is paramount to helping areas where the number of cases surpasses health-care system capacity. The first step to determine if elective and less urgent surgeries can resume is whether the local community has enough PPE. Communication about inventory, incoming supply chain, local case counts, and trends all should be considered before moving forward with any elective procedures. If the local government has indicated the need for redistribution of essential resources, such as PPE, due to scarcity, those orders must be followed.

### Tests

Currently, there are 2 forms of testing for the SARS-CoV-2 virus: polymerase chain reaction (PCR) and serology. PCR was invented in 1983 in Emeryville, California, and has been used for decades.^11^ It involves determining a unique segment of nucleic acids of the virus and creating a reagent that selects for that specific segment of RNA of the virus. The attachment is amplified and detected, thus providing a positive test. The test will only be positive if an individual is shedding the virus, and if the culture is performed appropriately. For example, if the swab does not appropriately brush the nidus of the virus, a false negative result may occur.

Serology testing is where one has blood drawn, and antibodies to the virus are detected using methods such as the enzyme-linked immunoabsorbent assay (ELISA). Positive results of immunoglobulin (Ig) M can indicate active infection in a patient, although the significance of IgG positivity remains uncertain. Seroconversion can take anywhere from 11-12 d postinfection; thus serology testing can lead to false negative results within 2 wk of initial innoculation.^12^ Additionally, immunosuppressed patients may fail to produce an adequate antibody response. Serology testing thus has numerous limitations that should be considered before widespread use.

### Testing Workflow

Patients are notified and screened by means of telephone and email the day before about the testing protocol. If they are unwilling to be tested, they forfeit their opportunity for elective surgery. Patients are informed that only they will be allowed in the facility. Any aid in mobility or language interpretation will be done by means of the nursing staff of the ASC. A screening update regarding symptoms (eg, cough, shortness of breath, fever, etc.) and contacts is performed. If any of the screening questions are concerning for COVID-19, the patient is advised to postpone. Patients are advised that measures are taken to reduce the risk of acquiring SARS-CoV-2 but that the possibility of transmission would still exist and would sign consent to proceed with surgery in light of this risk.

The creation of a CFZ requires remote outdoor testing, similar to what was implemented in South Korea for testing. Vehicles would enter a specific location with the health-care workers fully donned in PPE (airborne, droplet, and contact precautions). Alternate arrangements (eg, walk up zone) can be made in advance for testing of those patients without access to vehicles. The patient would be identified with the proper identification and would consent to testing and how the results would be shared with the county Department of Public Health (DPH). The health-care staff would first implement a nasopharyngeal swab sample. The swab would be directed to a PCR with a high positive percentage agreement (PPA). Because there is no standard lab test for the detection of SARS-COV-2, the exact sensitivity and specificity of each test is unknown. However, as per the data submitted to the FDA under the emergency use authorization (EUA) paperwork, certain PCR test kits have a PPA of 95% or above. This would correspond to an approximate false negative rate of 5% or below.

**Figure 1.**
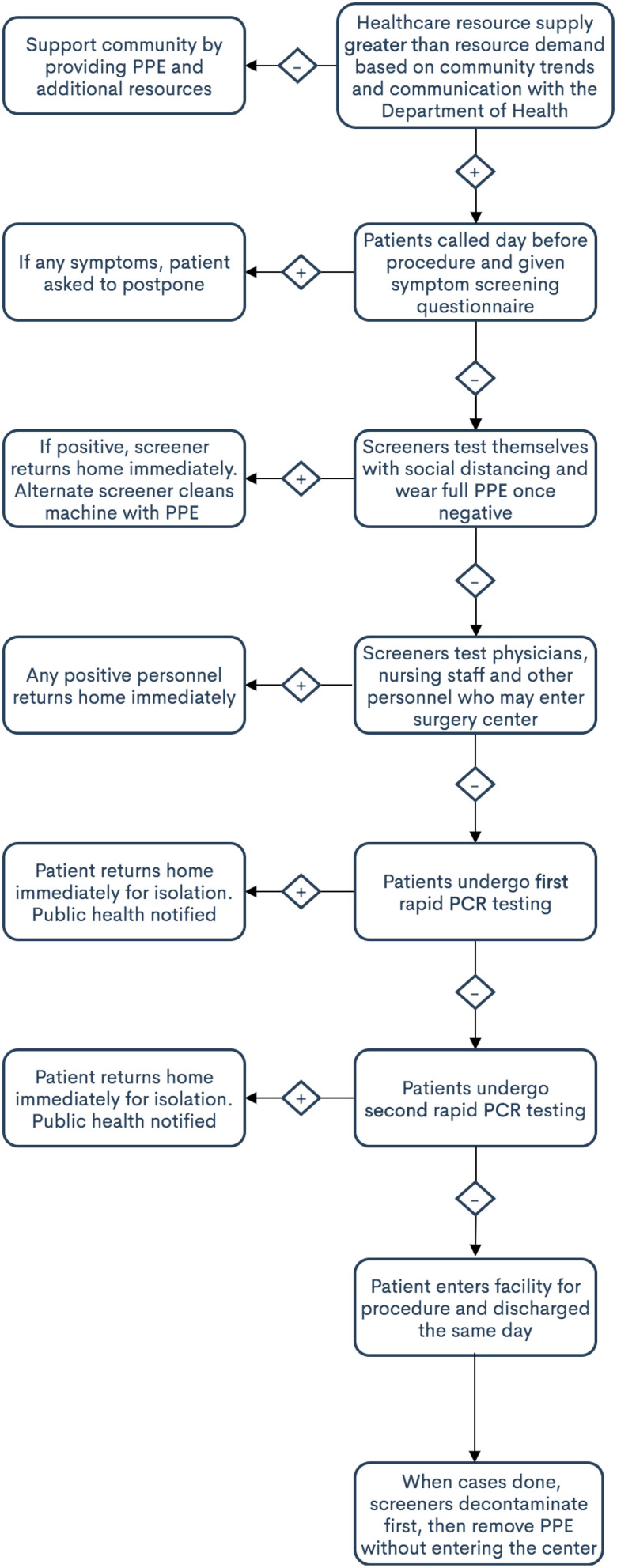
Primary testing workflow to open ambulatory surgery centers after the COVID-19 pandemic.

**Figure 2.**
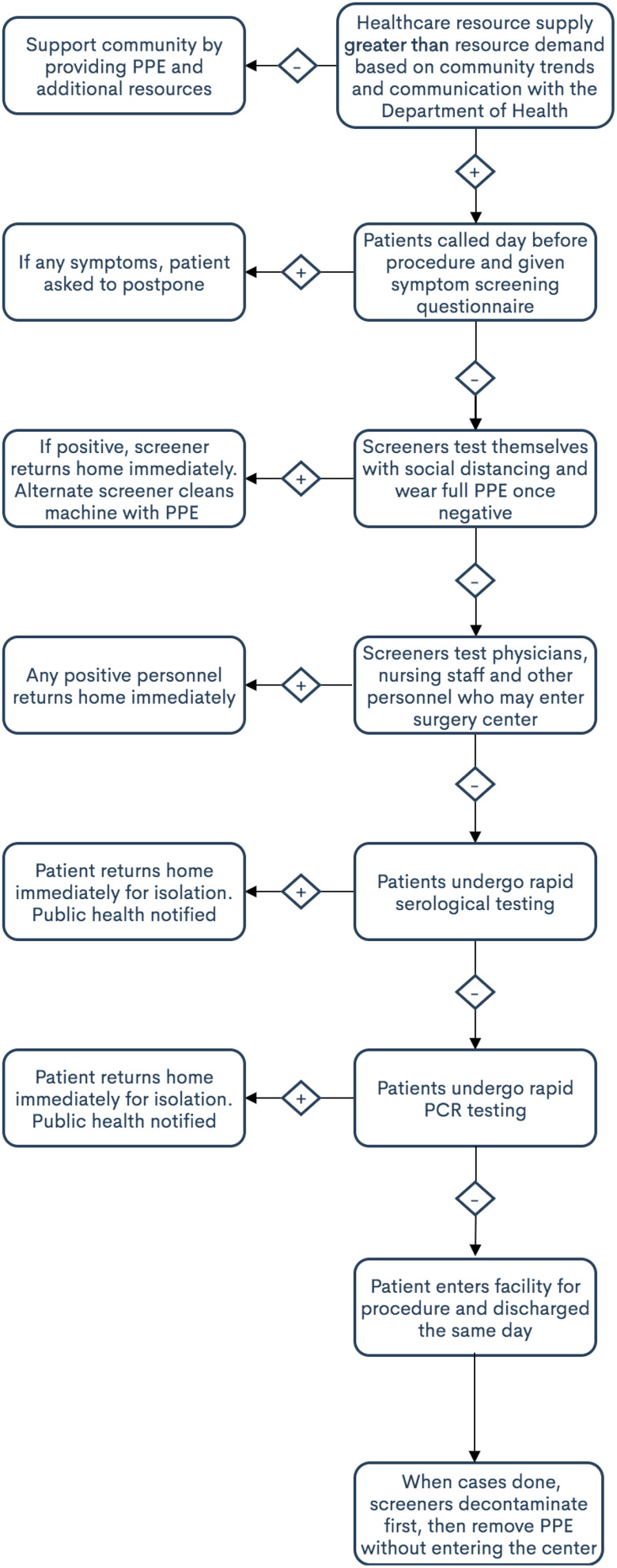
Secondary testing workflow to open ambulatory surgery centers after the COVID-19 pandemic.

However, this would mean that for every 20 patients, 1 might actually be positive for COVID-19. Given the limited PPA information available, the authors advise a second PCR test to reduce the false negative rate to well below 1% (Figures 1 and 2). Until further data come out from the widespread testing results of the current PCR tests, a second PCR test can help to ensure a lower overall false negative rate. A secondary approach would be to implement a serology test followed by a rapid PCR test, although this is certainly less ideal given the uncertainty with serology testing as described above. Indeed, a negative PCR test with a positive IgG may provide some information as to possible recovery of a patient, although that is not completely clear at this time. The patient would be asked to wait at a specific parking spot and patients would not be allowed to leave their vehicles until the results return. All screening staff within 6 feet of the vehicle would require appropriate PPE. We envision test results taking anywhere from 10 to 40 min, depending on whether a single or dual test is performed.^13^

If an individual is deemed *positive*, he or she will be provided information on what to do if symptoms limit their respiratory function, and how to seek help. This would depend on the community’s protocol for hospitalization. Positive individuals are advised to return home with mandatory 14 d of isolation from their friends and family members. The county DPH will be notified about the individual, including demographic information to aid in the understanding of this virus. The health-care workers involved in testing will need to be decontaminated ± doff and don new PPE. Protocols would be subject to change.

If an individual is deemed *negative* after the entire protocol, they are given signage that reflects this result, and they may enter the facility by means of 1 designated entrance. The other members of the vehicle are asked to remain in the vehicle or leave the premises with their contact information obtained.

All individuals that will enter the ASC must be tested. This includes surgeons, nurses, techs, custodial services, vendors, repair/utility services, essential administrative staff, and patients. The first individuals to arrive at the center must be the test-screeners. They must self-test or test each other and await the results before entering the facility. As this is a time-consuming process, it is imperative that all patients arrive at least 1-2 h before their scheduled procedure/surgery to ensure an efficient ASC operation. Any individual that leaves the premises may not return. The rationale for this rule is that, if an individual leaves and picks up the virus at a different location (eg, the hospital), they will not test positive immediately.

### Use of PPE in the CFZ

Standard precautions are to be used once inside the CFZ. There is no need to use the PPE necessary for contact, droplet, and airborne precautions for facilities that continue to combat COVID-19, unless required by the individual case (eg, for intubation). Staff members should use regular surgical masks and standard precautions, aside from procedures such as intubations.

### Decontamination Process

Fortunately, SARS-CoV-2 is destroyed relatively easily by the agents that are typically used in a health-care facility. The primary change would be to wipe down and decontaminate all surfaces, including door handles, stair rails, etc., from outside the facility and inside the facility on a periodic basis based on the EPA approved cleaning supplies list (eg, quaternary ammonium, hydrogen peroxide, chlorine dioxide, etc.).^14^

### Daily Debrief

At the end of each day, the test-screeners decontaminate first, then remove PPE without entering the CFZ. They may not enter the CFZ at any point. Screeners may become infected and would have a false negative result if they tested themselves to enter the facility. The screening leader calls the hospital administrators and provides an update on whether any individuals tested positive that day. Individuals who are not scheduled for surgeries are not allowed to be tested unless there is any immediate threat, such as if a person tests positive and is in the same vehicle.

### Postprocedural Follow-Up

It is advised that patients receive not only a postprocedural or postsurgical follow-up phone call regarding the procedure, but also be informed to call the ASC if any symptoms of COVID-19 arise (eg, dry cough, fever, shortness of breath, nausea, vomiting, diarrhea, and myalgias). All patients should receive a phone call 14 d after their procedure to ensure that they did not develop symptoms. This is done to help identify any false negatives.

### Optics of Performing Elective Surgeries

We understand that, even if we take all the precautionary measures to mitigate the risk of transmission of SARS-CoV-2, it may be deemed inappropriate to perform elective surgery as other parts of the United States may have shortages in specific equipment. For this reason, it is important to determine if there are dire needs for expendable equipment daily. Media coverage and social media must be managed appropriately. ASC staff and personnel should limit any media or social media during the COVID-19 crisis. Also, given the varying viral load across the country, our strategy can be used as certain parts of the country are opened before others.

### Advancing the Public Good

As a testing center, the ASC can be a very helpful resource for the county DPH and the international scientific community. Providing information on positive cases can be enlightening and akin to “random” testing in the community, as patients undergoing elective surgeries otherwise are a representative slice of the community’s population. Information from our testing should be made available to the DPH. Additionally, data gathered from the newly emerging PCR and serology tests can help establish more accurate sensitivity and specificity data for the public.

## Beyond Medicine

### Understanding the Socioeconomic Impact

The impact of COVID-19 extends far beyond that of medical surgery centers. As we flatten the curve, we must come up with effective strategies to re-open the country on a more widespread scale. If we fail to adopt an efficacious testing strategy, there will be great economic impacts on the world that cannot be overlooked. Researchers at the University of Chicago understood the loss of productivity that occurs with isolation policies alone and argued for widespread testing as a solution for gradual re-entry into normal day-to-day life.^15^ The World Bank conservatively estimates that the costs of pandemics can be as high as 1% of global gross domestic product (GDP), compared with 2% of GDP for climate change and provides compelling arguments on how to effectively invest in health security.^16^ The anticipated losses in the United Sates are estimated to be around $10-15 trillion dollars of GDP reduction.^17^ The cost of investing in aggressive and technologically advanced contact tracing, widespread testing (by means of reverse transcriptase-PCR and serology testing), and advanced isolation methods, however, is approximately $2-3 trillion dollars.^17^ Therefore, by establishing testing protocols that could be applied by local businesses to test their staff and customers, life can gradually begin to return to normalcy, with both the public’s health and overall economy’s interest in mind.

## Limitations

This rapid point of care testing will still depend on many important factors, such as availability, false negative rates, and cost. Given the rampant effect that COVID-19 has had on patient care and the economic impact it has had on hospitals, private practices, and surgery centers, the cost of testing may be warranted. Moreover, given the mortality rates of COVID-19, a careful risk/benefit and cost benefit analysis will likely be more than favorable. Therefore, availability and false negative rates are the most important concerns to adopt this model. Provisions should be taken by hospital systems and ASC’s to secure supplies and materials necessary for testing.

## Conclusions

Options are limited and the growing cases of patients waiting for elective cases will cause a tremendous burden on the already overworked health-care system. Transition from the American opioid epidemic to the COVID-19 pandemic could result in a second peak in opioid prescriptions and opioid-related deaths as many of the other modalities for pain management are constrained. The treatment of chronic pain patients by means of effective interventions will help to reduce this burden. Other specialties should also work to adopt this paradigm as elective cases will soon become nonelective (ie, – screening tests, routine biopsies, etc). Adopting extensive testing here will not only reduce anxiety and fear for those taking care of patients as well as for patients undergoing these procedures, but also will help to reduce unnecessary use of PPE and assist in epidemiological studies. In addition to supporting the healthcare industry, the model of extensive testing is supported from an economic standpoint as well.^17^ Effective testing and isolation will safely allow patients to receive healthcare and will also allow the United States and the global population to return to normalcy. Continued efforts for vaccine development, treatments aimed at lessening symptoms, as well as technologies dedicated to assist with contact tracing should be expedited. Strategic approaches based on rigorous testing methodologies will be essential for the future.
